# Laboratory Evaluation of the Alere q Point-of-Care System for Early Infant HIV Diagnosis

**DOI:** 10.1371/journal.pone.0152672

**Published:** 2016-03-31

**Authors:** Nei-yuan Hsiao, Lorna Dunning, Max Kroon, Landon Myer

**Affiliations:** 1 Division of Medical Virology, University of Cape Town, Cape Town, South Africa; 2 Division of Epidemiology & Biostatistics, School of Public Health and Family Medicine, University of Cape Town, Cape Town, South Africa; 3 Department of Neonatal Medicine, University of Cape Town, Cape Town, South Africa; George Washington University, UNITED STATES

## Abstract

**Introduction:**

Early infant diagnosis (EID) and prompt linkage to care are critical to minimise the high morbidity and mortality associated with infant HIV infection. Attrition in the “EID cascade” is common; however, point-of-care (POC) EID assays with same-day result could facilitate prompt linkage of HIV-infected infant to treatment. Despite a number of POC EID assays in development, few have been independently evaluated and data on new technologies are urgently needed to inform policy.

**Methods:**

We compared Alere q 1/2 Detect POC system laboratory test characteristics with the local standard of care (SOC), Roche CAP/CTM HIV-1 qualitative PCR in an independent laboratory-based evaluation in Cape Town, South Africa. Routinely EID samples collected between November 2013 and September 2014 were each tested by both SOC and POC systems. Repeat testing was done to troubleshoot any discrepancy between POC and SOC results.

**Results:**

Overall, 1098 children with a median age of 47 days (IQR, 42–117) were included. Birth PCR (age <7 days) comprised of 8% (n = 92) tests while 56% (n = 620) of children tested as part of routine EID (ages 6–14 weeks). In the overall direct comparison, Alere q Detect achieved sensitivity of 95.5% (95% CI, 91.7–97.9%) and a specificity of 99.8% (95% CI, 99.1–100%). Following repeat testing of discordant samples and exclusion of any inconclusive results, the POC assay sensitivity and specificity were 96.9% (95% CI 93.4–98.9%) and 100% (lower 95% CI 98%) respectively. Among birth PCR tests the POC assay had slightly lower sensitivity (93.3% vs 96.5% in routine EID) and higher assay error rate (10% vs 5% in samples of older children, p = 0.04).

**Conclusion:**

Our results indicate this POC assay performs well for EID in the laboratory. The high specificity and thus high positive predictive value would suggest a positive POC result may be adequate for immediate infant ART initiation. While POC testing for EID may have particular utility for birth testing at delivery facilities, the lower sensitivity and error rate requires further attention, as does field implementation of POC EID technologies in other clinical care settings.

## Introduction

Perinatal HIV infection is associated with high levels of morbidity and mortality in the first year of life [1]. Early infant HIV diagnosis (EID) and prompt referral for treatment improves survival and subsequent development [2, 3] but access to EID is severely limited in many settings. Across most of the world the standard-of-care (SOC) for EID uses polymerase chain reaction (PCR) testing that typically requires costly equipment and highly skilled operators. In resource-limited settings, PCR testing for EID is therefore restricted to centralised specialised molecular laboratories located in major urban centres.

This specialised, centralised approach to EID testing creates a complex cascade of logistical steps between sampling, testing, return of results, infant follow-up, linkage to care and ART initiation [4]. The EID cascade not only prolongs the turnaround time for infant management but also leads to significant attrition at each step. In particular, adequate access of laboratory testing does not always result in linkage to care and ART initiation. In 2010, only 71% of PCR positive infants in South Africa were linked to subsequent ART service and for those who were linked to care and there was a median delay to treatment of 33 days [5]. This attrition is even more pronounced in other parts of the world, with 22%, 37% and 38% of HIV infected infant were initiated on ART in Senegal, Uganda and Cambodia, respectively [6]. In Botswana a review of 8 years of EID showed that only 41% of PCR positive infants children were alive and on antiretroviral therapy, 39% had died, and 20% were either lost to follow-up, had transferred, or their mothers declined ART [7]. Cumulatively, the barriers to laboratory-based EID testing mean that only an estimated 39% of HIV-exposed infants in the WHO EID priority countries underwent appropriate testing in 2013 [8].

Meanwhile, advances in prevention of mother-to-child transmission of HIV (PMTCT) during the past decade have resulted in both improved coverage [9] and more efficacious regimens [10] for PMTCT. These advances, particularly in coverage of antiretroviral therapy (ART) for HIV-infected mothers and antiretroviral prophylaxis for HIV-exposed infants, are reshaping EID in terms of optimal time of testing. Specifically, more potent perinatal PMTCT regimens have resulted in drops in HIV transmission risks both intrapartum and postpartum through breastfeeding; as the perinatal HIV transmission rate drops, there is an increasing focus on identifying intrauterine transmission with PCR at birth to further reduce infant mortality [11, 12]. In this context, the turnaround time from sampling to result reporting is becoming even more important as referral to care in the immediately postnatal setting may depend heavily on the rapid availability of PCR results.

Point-of-care (POC) technologies are widely considered as a possible solution to these kinds of programmatic challenges in delivering HIV prevention and treatment services. For example, rapid HIV antibody tests are recommended by the World Health Organisation (WHO) and used widely to diagnose adult HIV infection and initiate ART in resource-constrained settings [13], and point-of-care CD4 enumeration has enabled staging of new HIV diagnoses to improve linkage to care and initiation of ART in contexts with limited laboratory access [14]. In the case of EID, same-day, POC HIV PCR testing could reduce attrition and delays across the EID cascade by removing the time and logistical complexities related to specimen transport, testing, return of results and recall of patients. In turn, POC EID testing has the potential to greatly enhance infant treatment outcomes.

Several POC technologies for EID are being developed, but few are at an advanced stage of development or have been independently evaluated [15]. The Alere q HIV-1/2 Detect system (Alere Healthcare, Waltham, Massachusetts, USA) is a cartridge-based qualitative POC nucleic acid amplification test (NAT) that is designed for POC use and could be used in EID programmes. We evaluated the performance of the Alere q HIV-1/2 Detect system in a laboratory setting by comparing its result with the current EID standard of care in South Africa.

## Methods

### Design

We performed a laboratory-based evaluation of Alere q 1/2 Detect to compare the POC test performance with the local SOC system for EID PCR. Samples from HIV-exposed children under 2 years of age undergoing routine HIV PCR testing in Western Cape province of South Africa between December 2013 and August 2014 were used for this evaluation. Samples came from children enrolled in various levels of paediatric care ranging from routine EID programme in primary care clinics to neonates delivered at maternity hospitals and specialist paediatric services. During the study period, local PMTCT policies called for universal initiation of lifelong antiretroviral therapy (ART) for all HIV-infected pregnant women regardless of CD4 cell count or HIV diseases stage (per the WHO policy of “Option B+”)[16]. Infant HIV testing was called for routinely in infants at 6 weeks of age, with testing at birth conducted for infants considered to be at high risk of HIV infection. During the study period the overall transmission rate in the province was estimated at approximately 1% [17].

### Ethics and consent

The study was approved by the Human Research Ethics Committee of University of Cape Town Faculty of Health Sciences and the Provincial Government of the Western Cape. The laboratory evaluation of the POC system is done through testing of routine clinical sample. A waiver for written informed consent was given because patient identity was delinked from the samples following the capture of relevant demographic information.

### Procedures

Following local practice, infant EDTA specimens (200–500μL) were collected through heel-prick or venepuncture at health care facilities and whole blood samples were transported the Groote Schuur Hospital laboratory of the National Health Laboratory Services (GSH-NHLS) where routine EID PCR is conducted. Whole blood samples are transported and stored at 4°C and tested within 72 hours of blood draw. Dried blood spots (DBS) were not routinely used in the local EID testing and thus did not form part of this study. The SOC assay for the study was version 1 of Roche Cobas AmpliPrep /Cobas TaqMan (CAP/CTM) HIV-1 qualitative assay (Roche diagnostics, Branchburg, New Jersey, USA), targeting the GAG and LTR region of HIV genome. The test was performed according to the manufacturer’s instructions. The lower limit of detection of the assay is 20 copies/ml. Due to local concerns around previously published reports of false positivity on this assay [17], the laboratory treats a subset of CAP/CTM positive result with cycle threshold (CT) values >33 as inconclusive as part of standard operating procedures.

During the study period the Alere q HIV 1/2 Detect (the POC assay) was performed on 6 different Alere q instruments by 7 trained laboratory staff. For this, 25μl of whole blood was pipetted from the EDTA tube to the test cartridge. The test takes approximately 55 minutes to process. The POC result was considered valid when the cartridge built-in controls were passed by the instrument. When a quality control measure fails due to either sample of instrument errors, an error code was issued and this data was recorded by the laboratory staff. If sufficient sample was available, repeat testing of sample with POC assay error was performed.

### Sampling

The study sampling was conducted in two phases. First, consecutive specimens were tested on both the SOC and POC assay in parallel, with staff blinded to test results. Because of the relatively low prevalence of HIV in this context, a second phase of sampling was conducted with only positive SOC specimens referred for POC testing.

### Analysis

Data were analysed using Stata Version 12.0 (Stata Corporation, College Station, Texas, USA). In analysis, qualitative output of SOC (CAP/CTM) was used in the comparison against the POC assay. Test performance for all infants, as well as stratified by infant age (with categories for newborn testing, conducted at <7 days of age, and routine testing, conducted at 6–14 weeks of age) and testing facility (comparing primary care clinics to specialist paediatric care), was estimated using sensitivity and specificity as well as positive and negative predictive values, with exact 95% confidence intervals (CI). In addition, cycle threshold (CT) values form the SOC assay were used as semi-quantitative measures analysed as medians with interquartile ranges (IQR). POC test error rates were estimated as proportion of total number of samples tested with exact 95% CI. Throughout, Fisher’s exact tests were used to compare proportions and rank-sum tests were used to compare medians; all statistical tests are 2-sided at alpha = 0.05.

**Fig 1:**
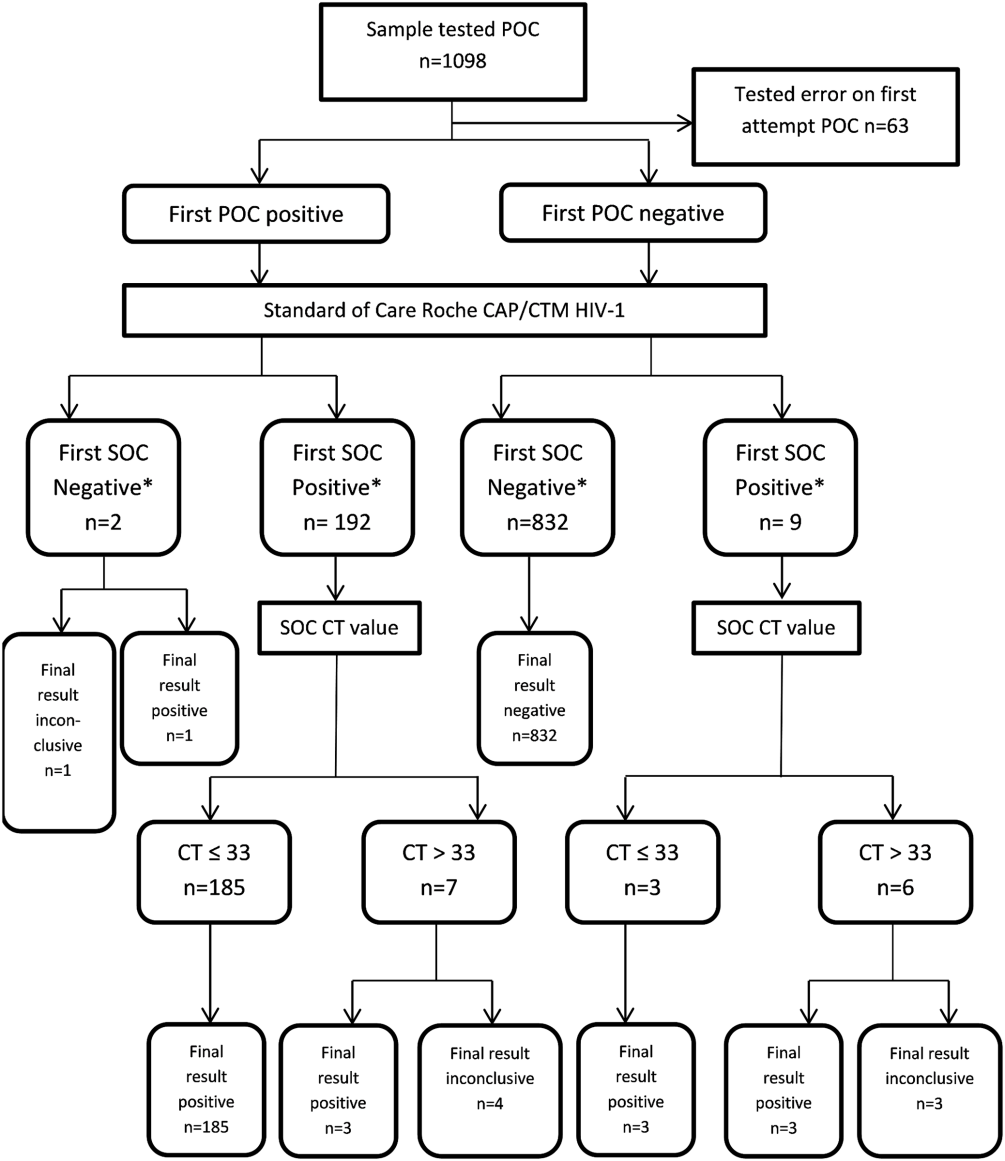
STARD diagnostic study flow diagram. The POC diagnostic accuracy analyses take into account 1) the result of first SOC test result* which is a direct comparison between the qualitative output of SOC and POC assay according to the manufacturer’s instructions. 2) Final test result which used the best result following retest for any inconclusive SOC result (CT > 33) and any discordant SOC/POC results. If an infant is conclusively positive on SOC assay in another sample, or positive on two different nucleic acid tests, the infant is considered positive in final result. Otherwise his/her final result is considered inconclusive.

Two sets of qualitative analyses are presented (Fig 1). The *first test result* is based on the first result produced by both the POC and SOC tests, irrespective of the CT value of the SOC result. In order to explore the discrepancies of the two assays and possible false positive SOC results, a *final test result* that allows for re-testing following inconclusive results was also analysed. In considering the final HIV test result, wherever the SOC result was reported as inconclusive or there was a disagreement between SOC and POC assays, further PCR testing on both platforms was performed pending suitable specimen availability. In the event of a first, inconclusive test result, a specimen is considered HIV-infected in the *final test result* when it tested positive subsequently on two different nucleic acid test assays or conclusively SOC assay positive on a different specimen.

This study report conforms to the STAndards for the Reporting of Diagnostic accuracy studies (STARD).

## Results

In total 1193 POC tests from 1131 samples were performed from 1098 children. The findings from the consecutive and positive-only sampling phases are identical, and reported jointly here. The demographic characteristics and SOC results for these children are summarised in Table 1. The median age of tested children was 47 days (IQR, 42–117) with 8% of children tested as newborns (age <7 days) and 56% of children tested as part of routine EID (ages 6–14 weeks). Children from primary clinic setting made up of 74% of the total children with 19% of children tested from a specialist paediatric service (n = 204). Overall, 212 of the 1098 (19%) children were positive according to the qualitative output of the SOC assay. However, 13/212 (6%) of these positive samples were interpreted as inconclusive based on CT<33.

**Table 1:** Summary study population demographic and facility characteristics by standard of care result status.

	SOC PCR	SOC PCR	
	Positive (%)	Negative (%)	Total
Total	212 (19%)	886 (81%)	1098
Median age	88 days	46 days	47 days
Age group			
<7 days	16 (17%)	76 (83%)	92
7 days to 6 weeks	14 (19%)	58 (81%)	72
6–14 weeks	81(13%)	539 (87%)	620
>14 weeks	101(32%)	213 (68%)	314
Sex			
male	88 (17%)	432 (83%)	520
female	117 (21%)	441 (79%)	558
unknown	7 (35%)	13 (65%)	20
Type of facility			
primary clinics	114 (14%)	698 (86%)	812
paediatric hospitals	83 (41%)	121 (59%)	204
maternity services	14 (17%)	67 (83%)	81

In the direct comparison on *first test results*, 1024/1035 (98.9%) of POC results were concordant with the SOC assay. Nine (0.8%) children tested SOC positive were negative for POC assay and two (0.2%) SOC negative samples tested positive on the POC assay (Table 2). The sensitivity of Alere q was 95.5% (95% CI 91.7–97.9%) and the specificity was 99.8% (95% CI 99.1–100%). The median CT value of SOC and POC positive samples was 24.3 (IQR 22.1–27.6), significantly lower than the median CT value of SOC positive, POC negative samples 34 (IQR 31.1–36.2 p<0.001). After resolving assay discrepancies and inconclusive SOC results with additional testing, the final test results were available for 1027 samples. Of these, 1021 final test results was correctly identified (99.4% concordance) with no false positive POC PCR were found while 6 false negative PCR were present, giving rise to sensitivity and specificity of 96.9% (95% CI 93.4–98.9%%) and 100% (lower 95% CI 98%) respectively (Table 3).

**Table 2:** Performance of Alere q Detect HIV-1 (first test) against the standard of care Roche CAP/CTM HIV-1 PCR.

	Roche CAP/CTM HIV-1 PCR		
Alere q Detect	Positive	Negative	Total
Positive	192	2	194
Negative	9	832	841
Total	201	834	1035

Compared to the qualitative CAP/CTM HIV-1 PCR result, the first attempt of Alere q achieved a sensitivity of 95.5% (95%CI 91.7–97.9%) with a positive Likelihood Ratio of 447.5. The specificity in this setting was 99.8% (95% CI 99.1–100%) with a negative Likelihood Ratio of 0.045.

**Table 3:** Performance of Alere q Detect HIV-1 in against different gold standards in various scenarios.

Comparison	n	Sensitivity (95%CI)	Specificity (95%CI)	LR+	LR-
Versus SOC	1035	95.5% (91.7–97.9%)	99.8% (99.1–100%)	447.5	0.045
Versus final result	1027	96.9% (93.4–98.9%)	100% (98%-100%)	N/A	0.031
Versus final result in routine EID	543	96.5% (87.8–99.5%)	100% (99.2%-100%)	N/A	0.035
Versus final result in newborn	81	93.3% (68.1–99.8%)	100% (94.6%-100%)	N/A	0.067
Versus final result in Specialist paediatric Hospital	181	97.3% (90.7–99.7%)	100% (96.6%-100%)	N/A	0.027

Of the samples tested, 6% (n = 67 samples from 63 children) of all first POC tests resulted in an error. In a subset of sample (n = 37) retested, 25/37 (68%) resolved after 1 additional test, 6/37 (16%) errors were resolved after 2 additional test and 6/37 (16%) gave repeated error messages despite 2 repeat attempts. Ninety percent of the recorded error codes were assay- or sample-related quality control failure. There were no associations between errors and a particular instrument or user.

**Fig 2:**
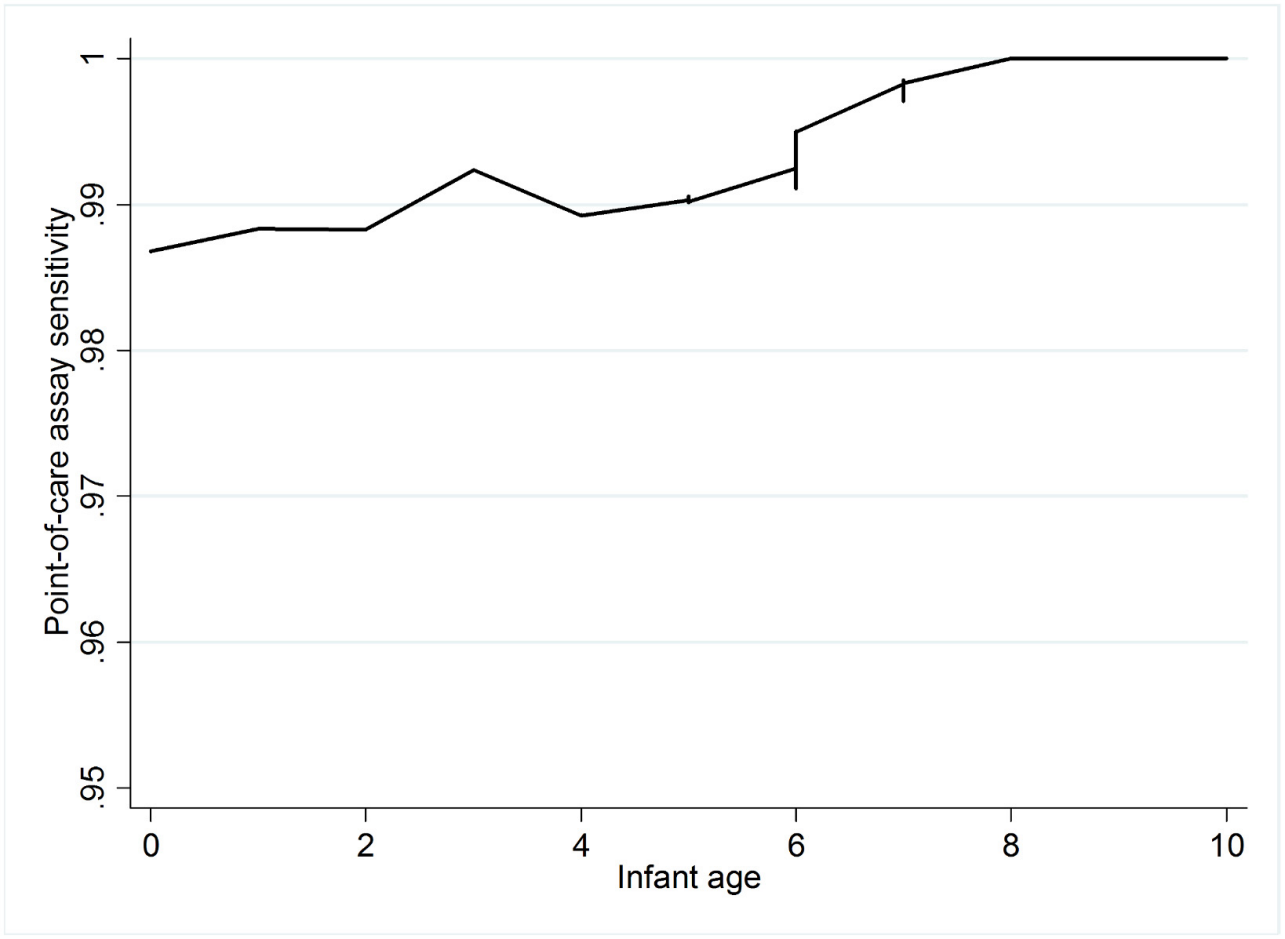
Lowess plot for sensitivity of infant testing in the first 10 weeks of life. The Lowess smoothing plot of POC sensitivity from infant samples across the age spectrum showed that the POC assay sensitivity is slightly lower in infants tested immediately after birth. The sensitivity improved as age of sampling increased. The optimal sensitivity was achieved around the routine EID age of 6–10 weeks.

To further evaluate the performance of the POC assay in the context of testing at birth, we stratified the comparison against final HIV result into three of the follow clinical scenarios: samples from newborns less than 7 days of age (n = 81), routine early infant diagnosis samples from primary care clinic between the age 6–14 weeks (n = 543) and samples from symptomatic infants of all ages from tertiary paediatric facilities (n = 181). The sensitivity of the assay was reduced to 93.3% (95%CI 68.1–99.8%; LR- 0.067) in the newborn stratum when compared to sensitivity of 96.5% (95%CI 87.8–99.5%; LR- 0.035) and 97.3% (95%CI 90.7–99.7%; LR- 0.027) in routine EID samples and symptomatic infants, respectively. A Lowess plot of test sensitivity across ages at testing in infant samples less or equal to 10 weeks of age (Fig 2) showed that the sensitivity of the assay appeared lower in the first 2 weeks of life, increasing until around 8 weeks. Assay errors were also more common from samples of the newborns category (10%) when compared with rest of the samples (6%, p = 0.04)

## Discussion

These novel data examine the performance of Alere q HIV1/2 Detect by comparing its result with a single current standard of care assay testing and after repeat testing. The latter is important given potential uncertainty around infant HIV diagnosis using the SOC assay in the context of maternal and/or child antiretroviral use [18,19, 20]. Our results showed that Alere q HIV 1/2 Detect showed excellent overall concordance with the SOC assay, with overall high sensitivity and specificity following repeat testing.

We found that the specificity of the test (99.8%) improved to 100%, with no false positive results detected, when the uncertainty of weakly positive SOC results were resolved through follow up testing. This small improvement may seem insignificant but in the era of maternal and infant antiretroviral use for treatment and/or prophylaxis, the incidence of MTCT is becoming extremely low making false positive results an increasing concern. In our setting (with an estimated infant HIV infection prevalence of approximately 1%) this increase corresponds to a substantial increase in positive predictive value from 84% to 100%. Thus, maximizing test specificity is an important feature of any EID platform, and the POC evaluated here performed well in this regard.

The sensitivity of any NAT is dependent in part on the volume of sample input. While the 25μl input for Alere q HIV1/2 Detect facilitates use for EID, this low sample volume and higher assay limit of detection may reduce test sensitivity compared to the SOC. The lower median CT observed here in false negative samples on the POC assay suggests that low copy number may be a key reason for the SOC positive sample unidentified by the POC assay. The detection of low copy numbers of HIV is a major challenge facing all EID assays, particularly in the era of ART prophylaxis for infants.

The reasons for discordant results between POC and SOC platforms require further investigation. Sequencing of HIV isolates in the assay target regions revealed that among the 11 discordant POC/SOC results, the 5’LTR region of one sample had two rare mutations which resulted in mismatches with the POC assay reporter (data not shown) which could have resulted in false negative POC results. In a second sample, HIV GAG sequences revealed three rare mutations that could have resulted in poor binding of SOC assay probe [21] and false negative SOC result.

Our study found 6% of samples resulted in assay error in laboratory setting. This is higher than the accepted error rate on our SOC platform (approximately 2–3%) and points to an important operational consideration in POC EID testing. Test errors due to failure of quality control or instrument failure could significantly affect the feasibility and costs of delivering EID via POC. We note that the vast majority of errors resolved upon retesting, and thus hypothesize that the errors may be due to specimen collection and/or operator errors. This issue needs to be thoroughly investigated in future field studies and a step-by-step trouble shooting guides for different types of errors should be developed prior the implementation of the assay.

There are some limitations of this study. The study was conducted in the laboratory and performed by laboratory staff that is familiar with NAT assays. This means several key questions around ease of use and performance in the field cannot be addressed. We did not have access to information on maternal ART use, nor on antiretroviral prophylaxis given to infants, and thus cannot comment on the potential role of antiretrovirals in influencing EID test results. Our study also did not include comparison against SOC assay performed on DBS, a common sample type for early infant diagnosis. As whole blood testing is more sensitive than DBS, we feel the comparison against DBS will likely be more favourable than our finding because the low copy target that is missed by the POC in this study may also be missed by the relatively insensitive DBS assays. The determinations of final infection status for some of the infants are incomplete due to limited blood samples received and the lack of follow up in many of these infants. Exclusion of these infants in the final HIV result could affect the analysis, although a small random group of infants is unlikely to significantly affect our estimate. Finally, we did not retest all the concordant results between the POC and SOC assay and thus any shared false positive/negative profile of the two assays would not have been detected. In order to know the true HIV infection status of infant in the POC assay evaluation, a large scale longitudinal study that include testing of subsequent samples and long term follow up would be required. Indeed, the 2014 WHO guideline of new strategy for diagnosing HIV infection among infants [22] highlighted that more thorough evaluation of virological testing in the setting of robust maternal combination antiretroviral regimens, prolonged infant antiviral prophylaxis and improved sensitivity of current HIV DNA- and RNA-based polymerase chain reaction (PCR) assays. With limited resources, low mother-to-child transmission rate and frequent lost to follow up; this is an important challenge facing all EID assay evaluations.

In conclusion, this study suggests that the Alere q HIV1/2 Detect performed well in a challenging population with high coverage of maternal ART and infant prophylaxis. The high specificity of Alere q HIV 1/2 Detect is a very important attribute for POC assays as it provide confidence for early ART initiation. Although its sensitivity is slightly lower in newborns, the assay’s performance and potential for POC use hold promise for EID implementation.

## Supporting Information

S1 File Database of study data. This database contains basic study data of all samples tested on point-of-care assay (Alere q Detect) and standard of care assay (Roche CAP/CTM). In addition to the qualitative results of both assays, the error code and cycle threshold value were captured for POC assay failure samples and SOC positive sample respectively. In some cases, individuals or samples were tested more than once due to either assay failure in the first attempt or discrepancy between POC and SOC assays. The repeat testing is captured under time tested column as 2^nd^ 3^rd^ 4^th^ or 5^th^ retests. Only first attempts were included in the primary analysis of the study.
(XLS)
